# Engineered kinesin motor proteins amenable to small-molecule inhibition

**DOI:** 10.1038/ncomms11159

**Published:** 2016-04-05

**Authors:** Martin F. Engelke, Michael Winding, Yang Yue, Shankar Shastry, Federico Teloni, Sanjay Reddy, T. Lynne Blasius, Pushpanjali Soppina, William O. Hancock, Vladimir I. Gelfand, Kristen J. Verhey

**Affiliations:** 1 Department of Cell and Developmental Biology, University of Michigan, 109 Zina Pitcher Place, Ann Arbor, Michigan 48109, USA; 2 Department of Cell and Molecular Biology, Feinberg School of Medicine, Northwestern University, Chicago, Illinois 60611, USA; 3 Department of Biomedical Engineering, Penn State University, University Park, Pennsylvania 16802, USA

## Abstract

The human genome encodes 45 kinesin motor proteins that drive cell division, cell motility, intracellular trafficking and ciliary function. Determining the cellular function of each kinesin would benefit from specific small-molecule inhibitors. However, screens have yielded only a few specific inhibitors. Here we present a novel chemical-genetic approach to engineer kinesin motors that can carry out the function of the wild-type motor yet can also be efficiently inhibited by small, cell-permeable molecules. Using kinesin-1 as a prototype, we develop two independent strategies to generate inhibitable motors, and characterize the resulting inhibition in single-molecule assays and in cells. We further apply these two strategies to create analogously inhibitable kinesin-3 motors. These inhibitable motors will be of great utility to study the functions of specific kinesins in a dynamic manner in cells and animals. Furthermore, these strategies can be used to generate inhibitable versions of any motor protein of interest.

## Abstract (web summary)

The use of specific small molecule inhibitors can contribute to the study of kinesins' cellular functions. Here the authors develop a chemical-genetic approach to engineer kinesin motors that can be efficiently inhibited upon addition of cell-permeable molecules.

## Graphical abstract

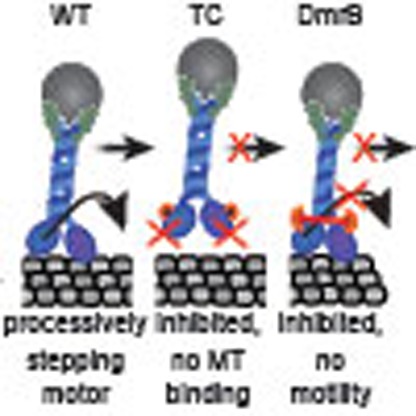

Microtubules are cytoskeletal filaments required for cell division, cell motility and intracellular trafficking and organization. Two motor protein families, kinesins and dyneins, produce force and motility along microtubule polymers, and defects in these motors are associated with human pathologies including neurodegeneration, tumorigenesis, developmental defects and ciliopathies^1^,^2^,^3^,^4^. Kinesins contain a highly conserved ∼350 amino-acid kinesin motor domain with signature sequences for ATP hydrolysis and microtubule binding. Many kinesins undergo processive motility and advance along the microtubule surface as dimeric molecules by alternate stepping of the two motor domains^5^. Outside of the motor domain, each kinesin contains unique sequences for cargo binding and regulation, and thereby carries out specific cellular functions^6^,^7^.

Mammals contain ∼ 45 kinesin genes that are classified into 17 families based on phylogenetic analysis^8^. To identify the cellular roles of specific kinesin gene products, genetic approaches (for example, knockout animals) and classical protein inhibition methods (for example, RNA interference (RNAi), overexpression of dominant-negative proteins, injection of inhibitory antibodies) have been utilized. However, these approaches are hampered by off-target and indirect effects, gradual inhibition of the targeted kinesin, and/or the lack of temporal control of protein inhibition, and are thus not optimal for dissecting complex and dynamic cellular pathways. These drawbacks could in principle be overcome by the use of cell-permeable inhibitors, but screening efforts with small-molecule libraries have yielded only few specific inhibitors^9^; most inhibitors target multiple kinesin motors, presumably due to the high conservation of the kinesin motor domain^10^,^11^.

Here we report a ‘chemical-genetic' engineering approach to generate kinesin motors that are amenable to small-molecule inhibition. Using kinesin-1 as a prototype, we developed two independent strategies to engineer genetically modified motors that transport cellular cargoes in a manner indistinguishable from the wild-type (WT) motor but that can be rapidly and specifically inhibited with high specificity by the addition of a small molecule. Our approach enables investigation of the function of the kinesin-1 motor protein in cells or animals with high temporal resolution and specificity. Furthermore, we demonstrate that both strategies can be transferred to kinesin-3, which can be engineered in similar manner as kinesin-1 to yield inhibitable motors. Based on the high conservation of the motor domain across the kinesin superfamily and the development of two different inhibition strategies, we suggest that these strategies can be used to generate inhibitable versions of any kinesin motor of interest.

## Results

### Designing kinesins amenable to small-molecule inhibition

Kinesins that are engineered to study motor function in cells and animals must fulfill two criteria. First, the engineered motor must maintain the microtubule-dependent motility properties of the WT protein and second, it must be specifically inhibited by a small, membrane-permeable molecule. Thus, a successful design will minimally alter the structure of the motor yet will mediate binding of the inhibitory molecule with high specificity and affinity. We pursued two strategies to yield kinesins that can be inhibited by addition of a small molecule. Both strategies were first implemented and tested with kinesin-1 because it is the best-characterized member of the kinesin family and assays to study its motility and function are well established (for example, refs 12, 13, 14, 15, 16, 17, 18, 19).

**Figure 1:**
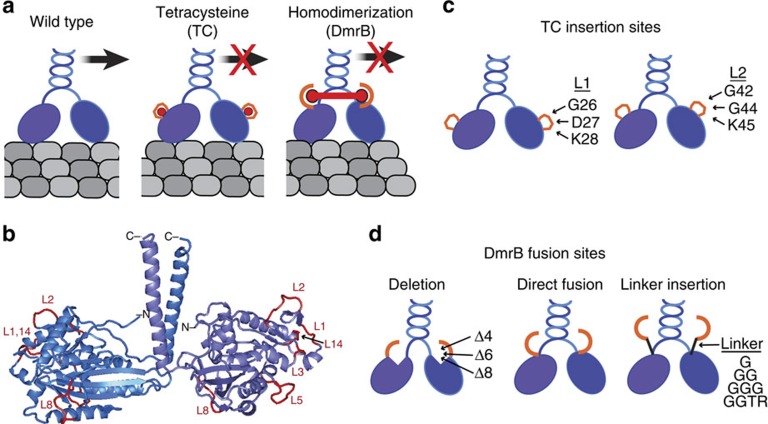
Strategies for engineering inhibitable kinesin motors. (**a**) The two motor domains (blue ovals) of a dimeric kinesin motor undergo alternating catalysis to generate processive motility along the microtubule surface (grey). The tetracysteine (TC) strategy inserts a TC tag (orange loop; sequence CCPGCC) into surface loops of the kinesin motor domain. Binding of the biarsenic dye ReAsH (red circle) to the TC tag is expected to restrict the conformational changes of the motor domain during catalysis and thereby inhibit processive motility. The homodimerization (DmrB) strategy fuses a DmrB domain (orange semicircle) to the N-terminus of the kinesin motor domain. Addition of the B/B homodimerizer (red dumbbell) to the DmrB domains crosslinks the motor domains and is thereby expected to inhibit processive motility. (**b**) Ribbon diagram of a dimeric kinesin-1 motor domain (PDB 3KIN) showing surface loops tested for TC insertion using the *Dm*KHC(1-560) construct. (**c**,**d**) Schematic of engineered *Rn*KIF5C(1-559) kinesin-1 constructs. (**c**) The TC motif was inserted into loop L1 (G26,D27,K28) or L2 (G42,G44,K45). (**d**) The DmrB domain was fused directly to the N-terminal methionine (middle), extended from the motor domain by short peptide linkers (G, GG, GGG or GGTR, right) or brought closer to the core motor domain by deletion of the N-terminal 4, 6 or 8 amino acids (Δ4, Δ6, Δ8, left).

Our first strategy for engineering inhibitable kinesin-1 motors took advantage of the ability of membrane-permeable biarsenical dyes (FlAsH and ReAsH) to bind to the small tetracysteine tag (TC, amino-acid sequence CCPGCC) and thereby label TC-tagged proteins in live cells^20^,^21^. We hypothesized that when the TC tag is inserted into the surface of the kinesin motor domain it will, in a ligand-dependent manner, restrict the conformational changes that occur during the catalytic cycle and thereby inhibit the motor (Fig. 1a). This strategy was first tested using a truncated and active version of the *Drosophila melanogaster* kinesin heavy chain motor (*Dm*KHC(1-560)). The motility of motors containing TC tags in six different surface loops (L1, L2, L3, L5, L8, L14; Fig. 1b and Supplementary Fig. 1a) was examined at the population level using microtubule gliding assays. Insertion of the TC tag into L3, L5 or L8 abolished motor activity (Table 1) and modification of these loops was not pursued further. Insertion of the TC tag into L14 resulted in motors with reduced gliding speed that was not altered by the presence of the FlAsH dye (Table 1) and modification of this loop was also not further pursued. Importantly, insertion of the TC tag into L1 or L2 resulted in motors that had microtubule gliding activity similar to that of the WT motor in the absence of inhibitor and no measurable gliding activity in the presence of the FlAsH dye (Table 1). We thus pursued the insertion of a TC motif into L1 or L2 for generating an inhibitable mammalian kinesin-1 motor using a truncated and constitutively active version of the *Rattus norvegicus* kinesin-1 motor *Rn*KIF5C(1-559) (Fig. 1b,c and Supplementary Fig. 1b,c).

Our second strategy to engineer inhibitable kinesin-1 motors was based on the ability of the cell-permeable rapamycin analogue B/B homodimerizer (Rapalog-2 or AP20187) to induce homodimerization of the DmrB domain (F36V variant of FKBP^22^,^23^). We reasoned that fusion of the DmrB domain to the N-terminus of the kinesin motor domain will create a situation in which addition of B/B homodimerizer (hereafter called B/B) crosslinks the motor domains of the kinesin dimer and inhibits motor stepping (Fig. 1a). We created a series of constructs in which the DmrB domain was fused directly to the N-terminus of *Rn*KIF5C(1-559), separated from the N-terminal methionine by one (DmrB-G), two (DmrB-GG), three (DmrB-GGG) or four (DmrB-GGTR) amino acids, or situated closer to the core motor domain by removing the N-terminal four (DmrBΔ4), six (DmrBΔ6) or eight (DmrBΔ8) amino acids of KIF5C (Fig. 1d).

### Initial analysis of engineered mammalian kinesin-1 motors

We first verified that the engineered TC and DmrB *Rn*KIF5C(1-559) motors are soluble when expressed in cells and retain microtubule-dependent motility in the absence of the inhibitors. To do this, we expressed the constructs in differentiated CAD cells where the defined microtubule architecture of the neuronal processes enables the accumulation of active kinesin motors at the distal tip^24^. All TC constructs showed accumulation in neurite tips (Supplementary Fig. 2), suggesting that insertion of the TC motif into L1 or L2 does not interfere with microtubule-dependent motility. However, TC(G44) did not express well in all cells and showed less neurite tip accumulation as compared with the WT motor (Supplementary Fig. 2) and was therefore excluded from further analysis. For the DmrB constructs, fusion of the DmrB domain directly to the N-terminus or separating it from the N-terminus via short linkers resulted in motor accumulation at neurite tips (Supplementary Fig. 2). However, positioning the DmrB domain closer to the core motor domain reduced or abolished tip accumulation (Supplementary Fig. 2), consistent with reports that these N-terminal residues form a cover strand essential for motor force generation^25^,^26^. The DmrBΔ4, DmrBΔ6 and DmrBΔ8 constructs were thus excluded from further analysis. All remaining TC and DmrB constructs generate stable proteins with the expected molecular weights (Supplementary Fig. 3) and were analysed further.

**Figure 2:**
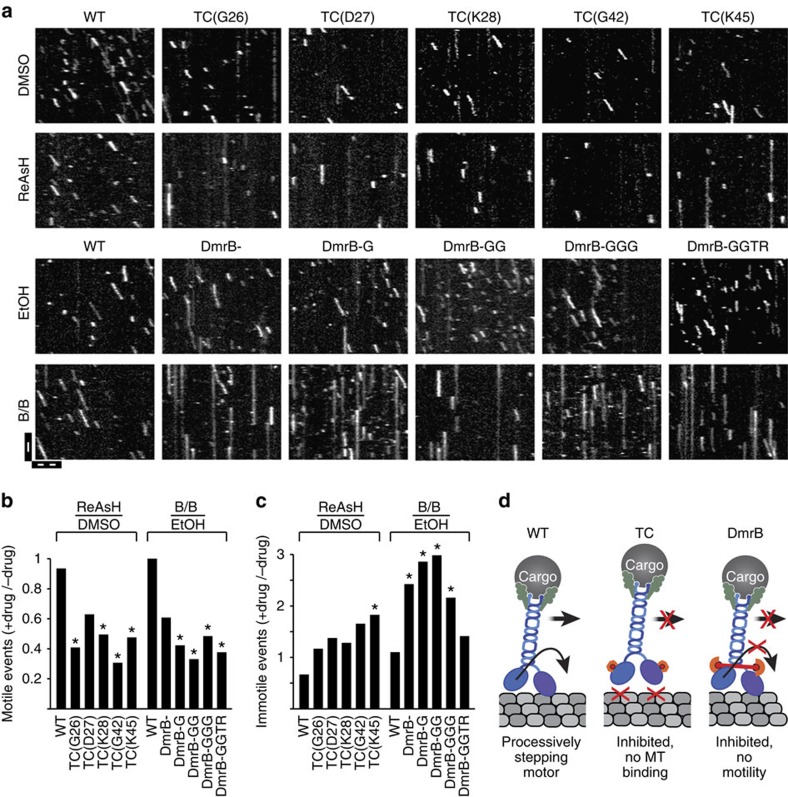
Inhibition of engineered kinesin-1 motors in single-molecule motility assays. (**a**–**c**) Lysates of COS7 cells expressing wild-type (WT), TC-tagged or DmrB-tagged *Rn*KIF5C(1-559)-3xmCit motors were incubated for 30 min in the absence (EtOH, DMSO) or presence (20 μM ReAsH, 1.5 μM B/B) of the respective inhibitor, and the motility of single motors along taxol-stabilized microtubules was observed by TIRF microscopy. (**a**) Representative kymographs. The vertical axis is time (scale bar, 3 s) and the horizontal axis is distance along the microtubule (scale bar, 5 μm). (**b**,**c**) The mean number of (**b**) motile and (**c**) immotile events per micrometre of microtubule per min was determined from seven to nine time-lapse movies over three independent experiments. The data are presented as a ratio of the (number of events +drug)/(number of events–drug). *P<0.05; heteroscedastic, two-tailed *t*-test comparing event frequency in the absence and presence of drug. (**d**) Proposed inhibition mechanisms for engineered kinesin-1 motors. (left) WT kinesin-1 moves processively along the microtubule surface (grey). (Middle) Binding of ReAsH (red circle) to the TC-tagged motor domain interferes with motility by inhibiting microtubule binding. (Right) Binding of B/B homodimerizer (red dumbbell) to the DmrB-tagged motor domain crosslinks the motor domains, resulting in microtubule binding but no motility.

To examine the activity of individual TC- and DmrB-tagged motors in the absence and presence of the inhibitors, we used total internal reflection fluorescence (TIRF) microscopy to image 3xmCit-tagged motors *in vitro* (Fig. 2a). For quantitative data analysis, we defined motile events as motors landing and processively moving (>250 nm) along the microtubule, whereas immotile events were defined as a motor landing and staying attached to the microtubule without detectable movement. Insertion of the TC tag into L1 or L2 caused a decrease in the number but not the velocity or run length of motile events (Supplementary Fig. 4a,c,d). Importantly, addition of ReAsH caused a significant reduction in the number of motile events (Fig. 2a,b and Supplementary Fig. 4a) and a small but generally not significant increase in the number of immotile events (Fig. 2a,c and Supplementary Fig. 4b). These results suggest that ReAsH-inhibited TC motors are unable to effectively engage with the microtubule track (Fig. 2d). Fusion of the DmrB domain to the N-terminus caused a small increase in the number of motile events but not their velocity or run length (Supplementary Fig. 4a,c,d). Addition of B/B homodimerizer resulted in a significant decrease in the number of motile events (Fig. 2a,b and Supplementary Fig. 4a) and a corresponding significant increase in the number of immotile events (Fig. 2a,c and Supplementary Fig. 4b), suggesting that B/B-inhibited DmrB motors can bind to the microtubule but cannot undergo processive motility (Fig. 2d).

For both TC- and DmrB-tagged motors, a fraction of the population was not inhibited and displayed similar motility parameters (run length and run velocity) in the absence and presence of inhibitor (Supplementary Fig. 4c,d). We thus chose constructs TC(G26) and DmrB-GG to probe the relationship between inhibitor concentration and motility in single-molecule assays. Increasing concentrations of ReAsH resulted in a corresponding decrease in the number of TC(G26) motile events (Supplementary Fig. 5a), supporting the assertion that ReAsH inhibits TC-tagged motors by blocking productive engagement of the motor with the microtubule (Fig. 2d). For the DmrB-tagged motor, an optimal concentration of B/B was observed; lower and higher concentrations were less effective (Supplementary Fig. 5e). We hypothesize that at high concentrations, different inhibitor molecules bind to each kinesin motor domain, reducing the crosslinking efficiency (Supplementary Fig. 5h).

In summary, our single-molecule analysis indicates that ReAsH interferes with the ability of TC-tagged motors to engage with the microtubule, whereas B/B interferes with the ability of DmrB-tagged motors to move processively along microtubules (Fig. 2d). For both strategies, inhibition is all-or-nothing, with no evidence of partial inhibition of motor activity (for example, decrease in velocity or run length).

### Transport mediated by engineered kinesins can be inhibited

**Figure 3:**
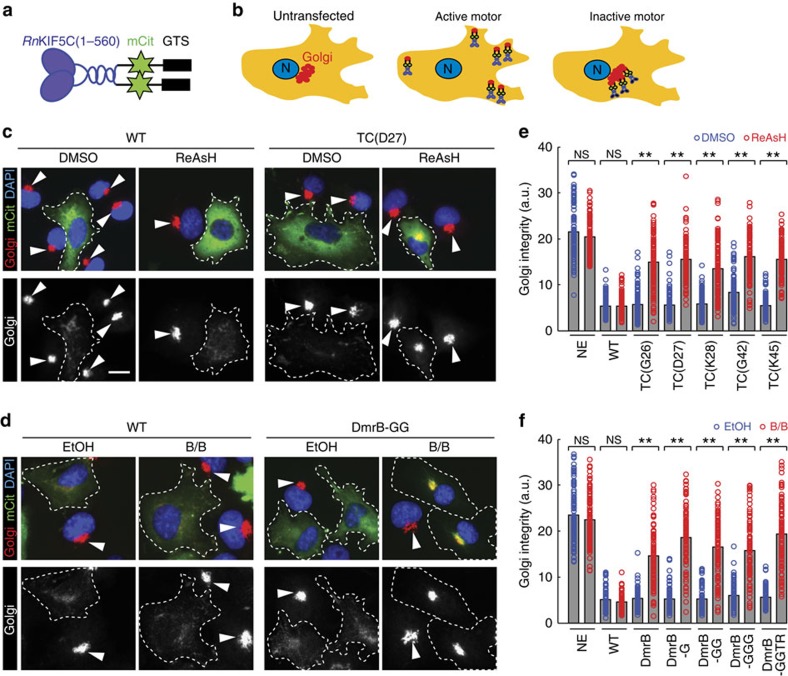
Inhibition of ectopic cargo transport by engineered kinesin-1 motors in cells. (**a**) Schematic of *Rn*KIF5C(1-559) motor tagged with mCit and a Golgi targeting sequence (GTS). (**b**) Schematic of Golgi dispersion assay. In untransfected cells (left), the Golgi complex (red) is localized in a tightly packed, perinuclear cluster. Expression of active kinesin-GTS motors (middle) results in dispersion of the Golgi complex, whereas inhibition of these motors (right) retains the tightly-packed, perinuclear Golgi localization. N, nucleus. (**c**–**f**) COS7 cells expressing WT, TC-tagged or DmrB-tagged motors with a GTS were incubated in the absence (DMSO or EtOH) or presence of inhibitor (200–400 nM ReAsH or 1.5 μM B/B), fixed ∼14 h later, and then stained with 4', 6-diamidino-2-phenylindole (DAPI; blue) and an antibody to a Golgi marker (Giantin; red). (**c**,**d**) Representative images of cells expressing (**c**) WT or TC(D27) constructs, or (**d**) WT or DmrB-GG constructs. The top panels show merged images, whereas the bottom panels show only the Golgi fluorescence signal. White dotted line, outline of cells expressing engineered motors. Arrowheads indicate compact Golgi complex in non-expressing (NE) cells. Scale bar, 15 μm. (**e**,**f**) Quantification of Golgi dispersion. The data are presented as the Golgi integrity where low values reflect a dispersed Golgi complex (motors active) and high values reflect a compact perinuclear Golgi complex (motors inhibited). Each circle represents the Golgi integrity of an individual cell in the presence (red) or absence (blue) of the respective inhibitor. Grey bars represent means of (**e**) *n*≥100 or (**f**) *n*≥76 cells over three independent experiments. *P*-values were calculated with a heteroscedastic, two-tailed *t*-test; NS, not significant (*P*>0.05); **P<0.000001.

Our overarching goal is to design engineered kinesin motors that possess WT function in cells but can be inhibited abruptly and specifically with a membrane-permeable small molecule. To characterize the inhibitable kinesin-1 motors in terms of cargo transport and inhibition in cells, we employed a Golgi dispersion assay^27^ in which Golgi-targeted active kinesin-1 motors cause the dispersion of the Golgi complex from its characteristic tightly-packed perinuclear location to a dispersed phenotype of small Golgi-derived vesicles scattered throughout the cytoplasm (Fig. 3a,b). For this assay, WT and engineered *Rn*KIF5C(1-559) motors were targeted to the Golgi complex by fusing these motors to the Golgi targeting sequence of the *cis*-Golgi resident protein GMAP210 (refs 27, 28, 29). Golgi-targeting of WT, TC-tagged and DmrB-tagged KIF5C(1-559) motors resulted in a dispersed Golgi phenotype (Fig. 3c–f), suggesting that the engineered TC and DmrB motors are not only capable of processive motility, but also generation of sufficient force to oppose the Golgi-localized dynein that is responsible for the perinuclear clustering of this organelle. Addition of ReAsH dye blocked the Golgi dispersion driven by TC-tagged motors but not WT motors (Fig. 3c,e). Likewise, addition of B/B blocked Golgi dispersion driven by DmrB-tagged but not WT kinesin-1 motors (Fig. 3d,f). These results demonstrate that the TC and DmrB kinesin-1 constructs are capable of cargo transport in cells and that this transport can be specifically inhibited upon addition of the relevant small-molecule inhibitor.

To determine the optimal inhibitor concentrations in cells, we performed dose–response experiments with constructs TC(D27) and DmrB-GG in the Golgi dispersion assay. For ReAsH, low micromolar concentrations caused nonspecific effects and 400 nM was determined to be optimal for TC-motor inhibition in this assay (Supplementary Fig. 6a). For the B/B homodimerizer, maximal inhibition was observed in the low micromolar range, similar to the effective concentrations used in other assays^30^, with no sign of nonspecific effects on WT motor constructs (Supplementary Fig. 6b).

**Figure 4:**
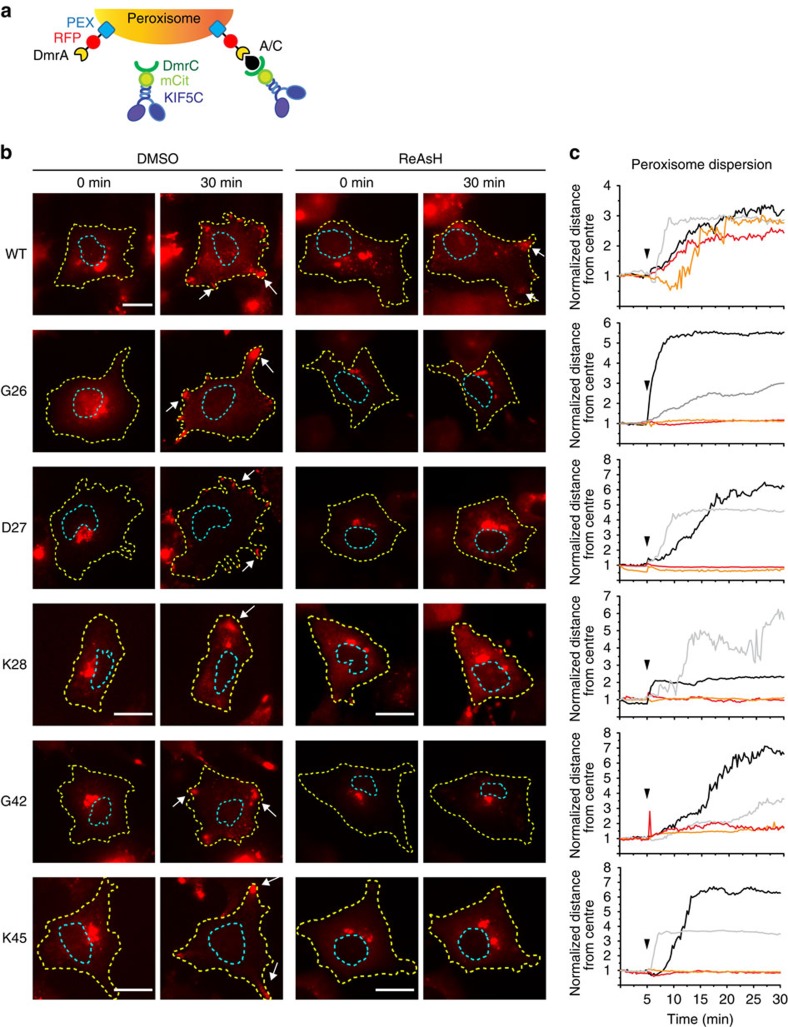
Rapid inhibition of TC-tagged kinesin-1-mediated peroxisome redistribution in cells. (**a**) Schematic diagram of the inducible peroxisome redistribution assay. WT- or TC-tagged *Rn*KIF5C(1–559) motor constructs fused to mCitrine (mCit) and a DmrC domain are recruited to the surface of peroxisomes containing a PEX-RFP-DmrA construct by addition of A/C heterodimerizer (or rapamycin). Recruitment of active motors results in redistribution of peroxisomes to the cell periphery. (**b**,**c**) Cells expressing the indicated WT or TC-tagged motor constructs were treated with DMSO (vehicle control) or 200 nM ReAsH for 30 min. Cells were then imaged for 30 min with rapamycin added 5 min after the start of imaging to induce motor recruitment to the peroxisome surface. (**b**) Representative images of peroxisome distribution at 0 and 30 min of rapamycin addition. Yellow dotted line, cell periphery; blue dotted line, nucleus. Arrows indicate the accumulation of peroxisomes in the periphery of the cell. Scale bar, 20 μm. (**c**) Representative time courses of peroxisome redistribution. Each line represents peroxisome movement in one cell. Black and grey lines, DMSO-treated control cells; red and orange lines, ReAsH-treated cells. The arrowhead indicates the time of rapamycin addition.

To gain a further understanding of the timescale of engineered motor inhibition, we used an inducible peroxisome redistribution assay^31^. For this assay, the DmrA (FKBP) domain is targeted to the peroxisome surface via a PEX sequence^32^ and rapamycin or A/C heterodimerizer (Rapalog-1 or AP21967) is used to recruit DmrC (FRB)-tagged kinesin motors to peroxisomes in a rapid manner (Fig. 4a). Hence, this assay provides a rapid readout of motor activity. Unfortunately, this assay could not be used to probe the DmrB constructs because of cross-reaction of the A/C heterodimerizer with the DmrB domain. Recruitment of WT or TC-tagged KIF5C(1-559) motors to the peroxisome surface in control dimethylsulphoxide (DMSO)-treated cells resulted in a redistribution of the peroxisomes to the cell periphery, as expected (Fig. 4b,c and Supplementary Fig. 7). Incubation of cells with ReAsH for 30 min had no effect on peroxisome redistribution driven by WT motors but abolished the redistribution driven by TC-tagged motors (Fig. 4b,c and Supplementary Fig. 7). These results demonstrate that addition of ReAsH efficiently inhibits the transport function of engineered TC motors within 30 min of inhibitor addition.

### Inhibitable kinesins rescue endogenous motor function

We next examined whether the engineered motors are able to rescue the function of the endogenous kinesin-1 motor in cells, and whether the transport of endogenous cargoes can be inhibited by the addition of the cognate inhibitor molecule. To do this, we chose *D. melanogaster* S2 cells because (i) expression of the single *Drosophila* kinesin-1 gene, *Dm*KHC, can be efficiently knocked down using double-stranded RNA (dsRNA) against the 3′ untranslated region (UTR) of the mRNA and (ii) *Dm*KHC is solely responsible for microtubule–microtubule sliding in these cells, thus providing a direct readout of kinesin-1 activity^33^.

We created full-length versions of the inhibitable, TC-tagged *Dm*KHC constructs G30 (L1) and G50 (L2; Table 1 and Supplementary Fig. 1a). We also created new DmrB-tagged versions of full-length *Dm*KHC based on our success with the DmrB-tagged *Rn*KIF5C(1-559) motors. As *Dm*KHC contains four additional amino acids on the N-terminus of its motor domain (Supplementary Fig. 1b), we fused the DmrB-domain directly to the N-terminus of *Dm*KHC (DmrB-*Dm*KHC, analogous to the *Rn*KIF5C(1-559) construct DmrB-GGTR), or we removed three amino acids from the DmKHC N-terminus (DmrB-Δ3*Dm*KHC, analogous to the *Rn*KIF5C(1-559) construct DmrB-G). To monitor expression, a blue fluorescent protein tag, mTagBFP2 (BFP)^34^, was fused to the C-terminus of each construct.

**Figure 5:**
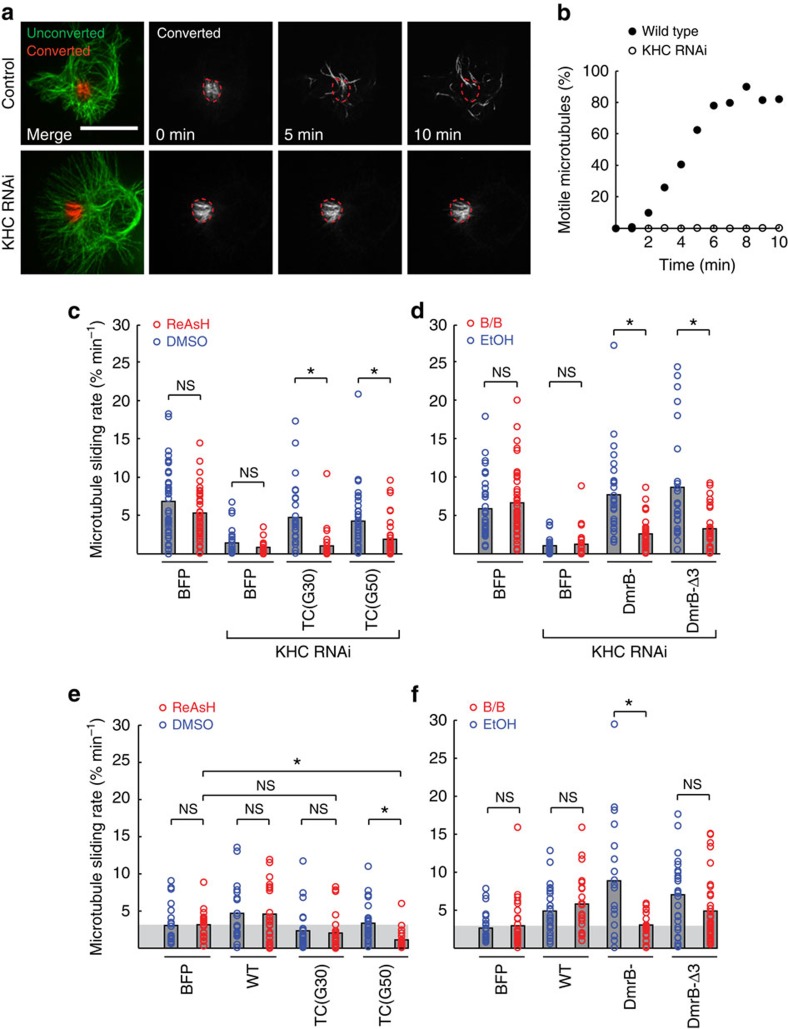
Inhibition of kinesin-1 function in *D. melanogaster* S2 cells expressing engineered *Dm*KHC motors. Microtubule sliding was measured in cells expressing tdEOS-tubulin under control (no RNAi), kinesin-1 knockdown (KHC RNAi) or upon knockdown and rescue with blue fluorescent protein (BFP) alone or BFP-tagged WT, TC or DmrB *Dm*KHC motors. On the day of imaging, cells were treated with vehicle (DMSO or EtOH) or inhibitor (200–400 nM ReAsH or 1.5 μM B/B) and tdEOS-tagged microtubules were photoconverted from green to red fluorescence in a region of interest. Photoconverted microtubules were tracked over a period of 5–10 min to measure microtubule sliding, a measure of kinesin-1 activity. (**a**) Representative images of microtubule sliding over time in control (no RNAi) or KHC RNAi-treated S2 cells. Scale bar, 15 μm. (**b**) Quantification of the microtubule sliding from **a**, showing the percentage of microtubules that moved out of the photoconverted region. (**c**–**f**) Microtubule sliding driven by engineered *Dm*KHC motors in (**c**,**d**) the absence of endogenous *Dm*KHC (KHC RNAi) or (**e**,**f**) the presence of endogenous *Dm*KHC. Each circle represents the microtubule sliding rate measured in a single cell. The grey bars indicate the mean of each condition (**c**–**d**: *n*≥25; **e**–**f**: *n*≥20 cells) from at least two independent experiments. High values reflect high *Dm*KHC activity, whereas low values reflect low *Dm*KHC activity. *P*-values were calculated with a two-sample Kolmogorov–Smirnov test; NS, not significant; *P<0.01.

Microtubule sliding was measured in S2 cells by expressing tubulin tagged with a tandem dimer of mEOS2 (tdEOS-tubulin)^18^,^35^, photoconverting an area of green fluorescent microtubules to red fluorescence, and measuring the movement of the red fluorescent microtubules over time (Fig. 5a,b). Knockdown of endogenous *Dm*KHC significantly reduced the microtubule sliding rate in comparison to control-treated cells (Fig. 5c,d), as reported previously^33^, and microtubule sliding was efficiently rescued by the ectopic expression of BFP-labelled TC-tagged or DmrB-tagged motors (Fig. 5c,d). Importantly, addition of ReAsH dye blocked the activity of the *Dm*KHC TC(G30) and TC(G50) motors as the microtubule sliding rate was reduced to that of the knockdown cells (Fig. 5c). Likewise, addition of B/B homodimerizer blocked the activity of the DmrB-*Dm*KHC and DmrB-Δ3*Dm*KHC motors (Fig. 5d). These results demonstrate that each of the engineered TC or DmrB *Dm*KHC constructs can rescue the activity of the WT motor and that microtubule sliding by the engineered motors can be efficiently inhibited by their respective inhibitors.

We then used microtubule sliding in S2 cells as an assay to test whether the engineered kinesin-1 motors can inhibit transport in the presence of the endogenous motor. In these assays, three types of kinesin-1 motors are generated in cells: homodimers containing two WT kinesin motor domains, heterodimers containing an engineered and a WT motor domain, and homodimers containing two engineered motor domains. Expression of *Dm*KHC TC(G30) surprisingly had a weak dominant-negative effect on endogenous kinesin-1 activity, but this effect is not significant and microtubule sliding was not further reduced by addition of ReAsH (Fig. 5e). In contrast, addition of ReAsH to cells expressing TC(G50) significantly reduced microtubule sliding relative to both vehicle-treated TC(G50) cells and to ReAsH-treated control (BFP-transfected) cells (Fig. 5e). This latter result suggests that kinesin motors with a TC motif engineered into L2 can inhibit the function of the endogenous motors *in trans*. For the DmrB-tagged motors, addition of B/B inhibitor reduced the microtubule sliding rate in cells expressing DmrB-*Dm*KHC and DmrB-Δ3*Dm*KHC but not below the level driven by the endogenous motor (Fig. 5f), suggesting that when inhibited, the DmrB motors do not interfere with the function of endogenous motors.

### Transfer of inhibition strategies to kinesin-3

The high sequence and structural conservation of the kinesin motor domain suggests that our chemical-genetic strategies can be used to engineer inhibitable versions of motors across the kinesin superfamily. To test this assertion, we applied the TC and DmrB inhibition strategies to the kinesin-3 motor KIF1A^36^,^37^,^38^ as we have experience with this motor^24^,^38^,^39^ and this motor possesses a well-characterized cellular function in transporting synaptic vesicle precursors in axons^36^,^40^. To test the transferability of the TC strategy, we generated a series of constructs in which the TC motif was inserted into different positions of L1 (TC(D23), TC(K25)) or L2 (TC(K41), TC(S51); Supplementary Fig. 8a) of a truncated and active version of the mammalian kinesin-3 motor *Rn*KIF1A(1-393) constitutively dimerized by a C-terminal leucine zipper (LZ)^38^. For the DmrB strategy, we engineered constructs in which the DmrB domain was fused directly to the N-terminus (DmrB-) or brought closer to the core motor domain by removing the N-terminal one (DmrBΔ1), two (DmrBΔ2), three (DmrBΔ3) or four (DmrBΔ4) amino acids. The function and inhibitability of these TC and DmrB constructs were characterized using the Golgi dispersion assay (Fig. 3b), as this assay probes the force generation and cargo transport function of an engineered motor in cells.

**Figure 6:**
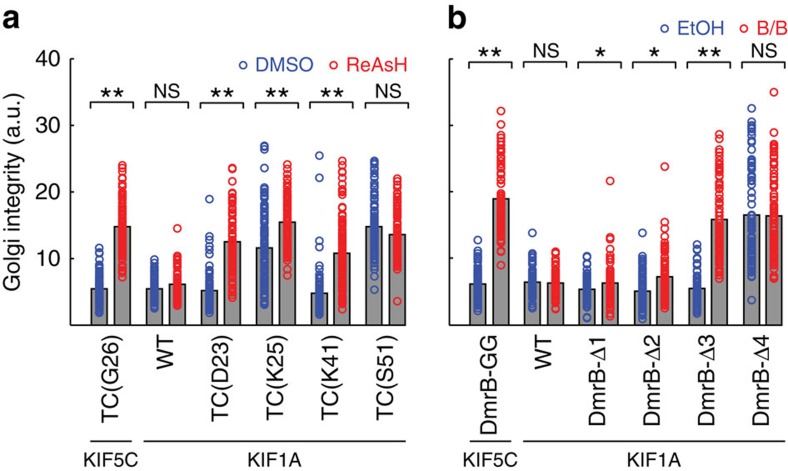
Inhibition strategies are transferable to kinesin-3 motors. TC-tagged and DmrB-tagged constructs were engineered for the kinesin-3 motor construct KIF1A(1-393)-LZ-mNG-GTS. The ability of WT, TC-tagged and DmrB-tagged motors to drive transport was measured in the Golgi dispersion assay. (**a**,**b**) COS7 cells expressing WT, TC-tagged or DmrB-tagged KIF1A motor constructs were treated with (**a**) 400 nM ReAsH or DMSO vehicle control or (**b**) 1.5 μM B/B or EtOH vehicle control. After 12 h, the cells were fixed and stained with 4', 6-diamidino-2-phenylindole (DAPI) and an antibody to a Golgi marker (Giantin), and the Golgi integrity was assessed as a measure of motor activity. Low values reflect a dispersed Golgi complex (motors active), whereas high values reflect a compact, perinuclear Golgi complex (motors inhibited). Each circle represents the Golgi integrity of an individual cell in the presence (red) or absence (blue) of the respective inhibitor. Grey bars represent means (**a**, *n*≥84; **b**, *n*≥74 cells) of three independent experiments. *P*-values were calculated with a heteroscedastic, two-tailed *t*-test; NS, not significant; *P<0.01; **P<0.000001.

In the absence of drug, the TC constructs TC(K25) and TC(S51) were unable to disperse the Golgi apparatus, indicating that engineering of the motor domain rendered these motors incapable of transport (Fig. 6a). Importantly, the TC constructs TC(D23) and TC(K41) were able to disperse the Golgi complex in the absence of drug, and this activity was abolished by addition of ReAsH to the cells (Fig. 6a and Supplementary Fig. 8b). Thus, engineering a TC motif into the kinesin-3 motor domain can generate inhibitable KIF1A motors. For the DmrB-tagged motors, the construct DmrBΔ4 was unable to disperse the Golgi apparatus in the absence of drug (Fig. 6b and Supplementary Fig. 8b), indicating that removal of this amino acid and/or addition of the DmrB domain at this position rendered the motor inactive. Importantly, the DmrBΔ3 construct was able to disperse the Golgi complex in the absence of drug and this activity was strongly inhibited by the addition of B/B homodimerizer (Fig. 6b and Supplementary Fig. 8b). Thus, engineering the DmrB domain onto the N-terminus of a kinesin-3 motor can also generate inhibitable KIF1A motors. Interestingly, although the constructs DmrBΔ2 and DmrBΔ1 were able to disperse the Golgi complex, these motors were not inhibitable (Fig. 6b), indicating that the position of the DmrB is critical for generating inhibitable motors. Taken together, these data demonstrate that the chemical-genetic inhibition strategies developed for kinesin-1 can be transferred to other members of the kinesin superfamily.

## Discussion

Chemical-genetic approaches have been successfully used to study the function of proteins for which the identification of specific inhibitors has remained elusive^41^,^42^. For ATPases such as kinases or motor proteins (including myosins and kinesins), such approaches have involved enlargement of the ATP-binding pocket to accommodate a nucleotide or inhibitor analogue that contains a bulky substituent complementing the enlarged pocket. For some kinases, modulation of the gatekeeper residue in the pocket resulted in diminished kinase activity and ATP affinity and recent efforts have focused on addressing this challenge^43^. Analogue-sensitive versions of kinesin-1 have been generated but have not been used for cell biological studies, as the engineered motor requires a modified ATP for full motor activity and the modified nucleotides are not membrane permeable^44^.

Here we describe a novel chemical-genetic approach to inhibit kinesins that has several advantages over existing inhibition methods. First, our approach enables rapid (within minutes) inhibition of motor function, in contrast to slow-acting methods such as knockout or knockdown that require days before motor depletion is achieved. Second, our approach uses known cell-permeable inhibitors that bind their target with high affinity and specificity, thus enabling selective and tunable inhibition of the targeted kinesin and avoiding off-target effects. Third, the engineered kinesins have properties similar to their WT counterparts, making the system ideal for acute inhibition across developmental time points. This feature is particularly advantageous for studying motor function when knockout or knockdown approaches result in a lethal phenotype. Fourth, the DmrB strategy is based on the established B/B homodimerizer (Rapalog-2), a chemical derivative of rapamycin with well-studied pharmacokinetics and the possibility of photocaging for enhanced spatial control in cellular systems^45^,^46^. Fifth, both of the described strategies (TC and DmrB), can be employed to generate inhibitable motors for different kinesin superfamily proteins, as demonstrated by successfully generating inhibitable motors for kinesin-1 from two different species (rat and fly) and two different rat kinesins (kinesin-1 and kinesin-3).

For the TC-tagged motors, addition of ReAsH reduced the number of motile events observed in *in vitro* assays. Thus, the major effect of the inhibitor is to reduce the effective engagement of the engineered motor with the microtubule track (Fig. 2d). This effect is likely irreversible as the ReAsH molecule binds covalently to TC tags^20^. For the DmrB-tagged motors, addition of B/B homodimerizer resulted in a dramatic increase in the number of immotile events. These immotile events have a limited lifetime, indicating that the inhibited kinesin does not adopt a rigor confirmation. Thus, B/B binding promotes, but does not trap, DmrB-tagged motors in a state of high microtubule affinity (Fig. 2d). Inhibition of DmrB-tagged motors is also likely to be irreversible as washout of the B/B ligand does not reverse DmrB homodimerization (for example, see ref. 31).

A reduction in the number of motile events for inhibited TC-tagged motors and a reduction in the processive motility for inhibited DmrB-tagged motors had a striking effect on cargo transport in cells. Indeed, both Golgi dispersion and microtubule sliding driven by kinesin-1 was nearly completely abolished by addition of ReAsH or B/B to cells expressing TC- or DmrB-tagged motors, respectively. The more dramatic effects of motor inhibition in cells than in *in vitro* assays may be due to experimental-specific conditions such as the dilute conditions of *in vitro* assays. Alternatively, it may be that force production during transport in cells renders cargo-bound motors more susceptible to inhibition. The dramatic and reproducible inhibition of the engineered motors in cellular assays is optimal for their envisioned use in studying the functions of kinesins in cells and animals. For future work with kinesin-1, we favour the constructs *Dm*KHC-TC(G30) and the corresponding rat KIF5C-TC(D27) because these constructs consistently performed well in all assays. For the DmrB-tagged fly and rat kinesin-1 motors, we slightly favour the rat construct DmrB-GG-KIF5C (which corresponds to DmrB-*Dm*KHC), because it shows the highest degree of inhibition across assays. To fully utilize the inhibitable kinesin-1 constructs, the engineered constructs should be expressed in cells in which endogenous kinesin-1 is either absent or has been depleted.

The high structural and mechanistic conservation within the kinesin motor domain has hindered the identification of kinesin-specific small-molecule inhibitors. However, this high conservation facilitates the direct transfer of the described chemical-genetic inhibition approaches to other members of the kinesin superfamily. Indeed, our results demonstrate that a fast and highly processive member of the kinesin-3 family can be engineered to be sensitive to inhibition by both ReAsH dye and B/B homodimerizer. It is interesting to note that while all of the DmrB-tagged kinesin-1 motors were equally inhibitable, only one out of four DmrB-tagged kinesin-3 motors gave rise to an inhibitable motor. This demonstrates the difficulty of predicting the precise position for optimal insertion of the TC or DmrB component. Thus, we recommend that the insertion position of the TC-tag or DmrB-domain be estimated based on X-ray structure and sequence alignment information and then a number of constructs be screened for an exploitable inhibitable motor. In our hands, the Golgi dispersion assay has worked well as a fairly rapid and quantitative assessment of the activity of engineered kinesin constructs.

In summary, we describe two general approaches that enable the rapid and specific perturbation of the cellular function of a kinesin of interest. These approaches will enable researchers to decipher the exact roles of different kinesins in various biological processes in specific cellular/developmental stages. We anticipate that our inhibition approaches will be applicable in cells and animals where an endogenous kinesin has been genetically replaced with its engineered counterpart. We also anticipate that these chemical-genetic strategies can be used to generate inhibitable myosin and dynein motors.

## Methods

### Plasmids and production of dsRNA

TC-tagged *Dm*KHC constructs for bacterial expression were generated using Splice by Overlap Extension PCR^47^,^48^ to insert sequences encoding a TC tag (CCPGCC) into L1 or L2 or two-step QuikChange PCR (XL II kit, Agilent) to introduce the TC tag into L3, L5, L8 or L14 of a *Dm*KHC(1-560)-eGFP-His6 construct. Primer sequences are found in Supplementary Table 1.

For mammalian kinesin-1, a truncated and active *Rn*KIF5C(1-559)-3xmCit^39^ construct was used. Sequences encoding TC- or DmrB-tagged KIF5C motor domain were synthesized (DNA2.0 or Life Technologies) and cloned in-frame. For the peroxisome redistribution assay, the last two mCits of the WT or TC-tagged motors were replaced by a DmrC domain (Clontech). The peroxisome anchor construct PEX3-mRFP-2xFKBP was a gift from Dr C.C. Hoogenraad (Utrecht University, Netherlands; ref. 49). For the Golgi dispersion assay, the Golgi-targeting sequence of *Hs*GMAP210 (amino acids 1757-1838, NP_004230) was synthesized (Life Technologies) and subcloned in-frame at the C-terminus of the WT or engineered kinesin-1 motors. For the kinesin-3 constructs, *Rn*KIF1A(1-393) with the leucine zipper dimerizing segment of GCN4 (ref. 38) was tagged at the C-terminus with mNeonGreen (Allele Biotechnology) followed by the Golgi-targeting sequence. Sequences encoding TC- and DmrB-engineered motor domains were synthesized (Life Technologies) and subcloned to replace the WT motor domain. For microtubule sliding assays in S2 cells, sequences encoding TC- or DmrB-tagged motors were synthesized (Life Technologies) and subcloned into the pMT/V5-His A vector (Invitrogen) containing full-length *Dm*KHC with an mTagBFP2 fluorescent protein at the C-terminus. All constructs were verified by analytical restriction digestion and sequencing.

Double-stranded RNA was generated by *in vitro* transcribing RNA strands with T7 polymerase followed by LiCl purification. Template DNA was generated by PCR of genomic DNA obtained from white-eyed (w1118) adult flies. The 5′ end of each primer used in PCR reactions contained the T7 promoter sequence (5′-TAATACGACTCACTATAGGG-3′). The annealing sequence of the forward KHC 3′-UTR primer is 5′-ATCCAATCACCACCTGTCGC-3′ and the sequence of the reverse is 5′-TCTGCGACTTTTATTTAGGT-3′.

### Cell culture techniques and immunofluorescence

COS7 cells (African green monkey kidney fibroblasts, American Type Culture Collection) were cultured in D-MEM (Gibco) with 10% Fetal Clone III (HyClone) and GlutaMAX (Gibco) at 37 °C and 5% CO_2_. CAD cells (mouse catecholaminergic cell line^50^) were cultured in D-MEM/F12 (Gibco) with 10% FCS and GlutaMAX at 37 °C and 5% CO_2_. *D. melanogaster* S2 (*Drosophila* Genomics Resource Center) cells were maintained in serum-free Insect-Xpress media with L-glutamine (Lonza). COS7 cells were transfected with Lipofectamine 2000 (Life Technologies), CAD with Trans-It LT1 (Mirus Bio LLC) and S2 cells with Effectene Transfection Reagent (Qiagen), each according to the manufacturer's instructions.

For single-molecule TIRF microscopy, COS7 cells expressing the fluorescently tagged motor constructs of interest were lysed 18 h post transfection. Cells were trypsinized, harvested by low-speed centrifugation, washed once in full culture medium and lysed in chilled Lysis Buffer (25 mM HEPES/KOH, pH 7.4, 115 mM potassium acetate, 5 mM sodium acetate, 5 mM MgCl_2_, 0.5 mM EGTA and 1% Triton X-100) supplemented with 1 mM phenylmethylsulphonyl fluoride and protease inhibitor cocktail (P8340, Sigma) and 1 mM ATP. The lysate was cleared by centrifugation at 16,000*g*, 4 °C for 10 min and aliquots were flash-frozen in liquid nitrogen and stored at −80 °C. The amount of motor proteins across lysates was determined by western (Supplementary Fig. 2) and dot blot analyses on BioTraceNT nitrocellulose membranes using monoclonal antibodies to GFP (1:3,000, A6455, Invitrogen) and kinesin-1 (1:5,000, MAB1614, Covance). ImageJ (NIH) was used for densitometric analysis.

For immunofluorescence, cells were fixed with 4% (vol/vol) formaldehyde in PBS, quenched with 50 mM NH_4_Cl in PBS, permeabilized with 0.2% Triton X-100 in PBS and blocked with blocking buffer (0.2% fish skin gelatin in PBS). Primary and secondary antibodies were applied in blocking buffer for 1 h at room temperature in the dark. Commercial antibodies used: polyclonal cis-Golgi marker giantin (1:500–1,500 PRB-114C, Covance), β-tubulin (1:2,000 clone E7; Developmental Studies Hybridoma Bank). Nuclei were stained with 4', 6-diamidino-2-phenylindole (final concentration 10.9 μM) and cover glasses were mounted in ProlongGold (Invitrogen). Images were collected on an inverted epifluorescence microscope (Nikon TE2000E) with a × 40, 0.75 numerical aperture (NA) objective and Photometrics CoolSnapHQ camera and analysed using ImageJ (NIH).

### CAD cell tip accumulation assay

CAD cells were seeded on cover glasses in growth medium, which was exchanged for serum-free medium 4 h later, followed by transfection. Two days later, cells were fixed and stained. Motor fluorescence at the proximal and distal ends of the neurites was measured and the tip accumulation is reported as the ratio of the distal (tip) fluorescence to the proximal (shaft) fluorescence.

### Golgi dispersion assay

COS7 cells were seeded on cover glasses and treated 4 h later with inhibitor (ReAsH-EDT2, Molecular Probes or B/B homodimerizer, Clontech) or its solvent (DMSO, Sigma, or ethanol, Decon Laboratories, Inc.) directly followed by the addition of transfection complexes. After 12–14 h, B/B-treated cells were fixed, whereas ReAsH-treated cells were first washed three times for 1 min each in BAL wash buffer (Molecular Probes) at room temperature and then fixed. For quantitative analysis of Golgi distribution, a MATLAB (Mathworks) script was developed to determine the standard deviation of pixel intensities (SDi) for the Golgi staining in the perinuclear region on a cell-by-cell basis. For this analysis, a region of interest representing the nucleus was manually selected, detected by a threshold-based method^51^, and then extended outward for 10 pixels. The SD_i_ in this dilated nuclear area was measured and then normalized to the square root of the mean pixel intensity (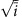) in the measured region. SD_i_ normalized with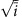 is not sensitive to different staining efficiencies and exposure times. Hence, the Golgi integrity parameter SD_i_/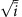 is well suited to quantitatively compare Golgi morphology across independent experiments. A high Golgi integrity value (SDi/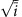) in the extended region of interest is obtained for a tightly packed, perinuclear Golgi, whereas a low Golgi integrity value is obtained for cells in which the Golgi-derived fluorescence signal is spread throughout the cell. For the DmrB-KIF5C constructs, inhibition was most efficient in cells expressing low levels of the engineered motor construct (based on mCit fluorescence, for example, see Fig. 3d).

### Inducible peroxisome redistribution assay

COS7 cells were plated onto glass-bottomed dishes (MatTek Corporation) and co-transfected 3 h later with plasmids coding for DmrC-tagged motors and the peroxisome anchor PEX3-mRFP-2xDmrA. Sixteen hours later, cells were washed with Opti-MEM (Gibco) and incubated for 30 min at 37 °C and 5% CO_2_ in Opti-MEM containing 0.1% DMSO (solvent control) or 200 nM ReAsH-EDT_2_ (Molecular Probes). Cells were then washed at room temperature three times for 1 min each with BAL wash buffer (Molecular Probes) and immersed in Leibovitz's L-15 medium (Gibco) supplemented with 200 μM Trolox (Sigma-Aldrich). Live-cell imaging was carried out at 37 °C in a temperature-controlled and humidified live-imaging chamber (Tokai Hit) on a Nikon Ti-E/B microscope equipped with a × 100, 1.49 NA oil immersion TIRF objective, three 20 mW diode lasers (488, 561, 640 nm) and electron-multiplying charge-coupled device (EMCCD) detector (iXon X3 DU897, Andor). Images were recorded in both the 488- and 561-nm channels every 15 s for 5 min and then 40 ng ml^−1^ rapamycin (Calbiochem) was added to the imaging medium and imaging resumed for another 25 min. Shifts in cell position during imaging were corrected with the Template Matching and Slice Alignment plugin to ImageJ (NIH) and time-lapse images were analysed with a custom MATLAB (Mathworks) script. First peroxisome objects were detected in each image in the 561-nm channel by a local adaptive thresholding algorithm (Guanglei Xiong; Tsinghua University; http://www.mathworks.com/matlabcentral/fileexchange/8647-local-adaptive-thresholding). Subsequently, the average distance of all peroxisome object pixels from a manually determined cell centre was reported for each frame to monitor peroxisome movement over time. In addition, the change of the average 488 nm fluorescence intensity over the peroxisome object pixels was determine to measure motor recruitment to peroxisomes. Distance and intensity graphs were generated using the Origin software (OriginLab).

### *In vitro* microtubule gliding assay

TC-*Dm*KHC motors were expressed in bacteria, purified and then incubated (∼10–20 μM) with 1 mM tris(2-carboxyethyl) phosphine for 5 min in elution buffer (50 mM NaPO_4_, 300 mM NaCl, 500 mM imidazole, 1 mM MgCl_2_, pH 7.0) to reduce the thiol groups in the cysteines. Reduced motors were then incubated with approximately fivefold excess TC-FlAsH II (catalogue no. T34561, Invitrogen) for 2 h at 4 °C. Protein concentration and labelling stoichiometry were measured using a ultraviolet absorption spectrophotometer. Taxol-stabilized Cy5-labelled microtubules were adsorbed onto the surface of flow cells, and the surfaces were blocked with 2 mg ml^−1^ casein. Motility solution consisting of ∼20 pM motors, 1 mM ATP, 0.2 mg ml^−1^ casein, 10 μM Taxol and an oxygen scavenging of 20 mM D-glucose, 0.02 mg ml^−1^ glucose oxidase, 0.008 mg ml^−1^ catalase and 0.5% (v/v) β-mercaptoethanol in BRB80 (80 mM PIPES, 1 mM MgCl_2_, 1 mM EGTA, pH 6.8) was then introduced. Microtubule gliding was visualized by TIRF using Nikon TE2000 microscope (× 60, 1.45 NA PlanApo) equipped with a 488-nm Ar ion laser for GFP excitation and a 633-nm He-Ne laser for Cy5 excitation; experiments were performed at 26 °C. Images were captured with a Cascade 512 CCD camera (Roper Scientific) and acquisition and image analysis carried out using MetaVue software (Molecular Devices Corporation); pixel size was 71.0 nm.

### Microtubule sliding assay in S2 cells

S2 cells were plated at 1 × 10^6^ per 1 ml media in 12-well dishes and co-transfected with tdEos-α-tubulin84B and BFP-tagged engineered kinesins (1 μg DNA total in a ratio of one to three). Immediately after transfection, 18 μg of dsRNA targeting the 3′-UTR of KHC was added directly to the media. After 48 h, another 18 μg of dsRNA was added along with 200 μM copper sulfate to induce expression of the transfected constructs. Cells were imaged ∼96 h after initial transfection and dsRNA treatment. On the day of imaging, transfected S2 cells were plated on Concanavalin-A-treated coverslips 10 min before addition of either 0.3% EtOH/1.5 μM B-B Homodimerizer (Clontech) or 0.1% DMSO/400 nM ReAsH (Invitrogen) for 30 min. Cells treated with ReAsH were washed three times with 1 ml 1 × BAL wash buffer in Insect-Xpress media. Before imaging microtubule sliding, cells were treated with 2.5 μM Cytochalasin D for 10 min at room temperature to depolymerize F-actin. Subsequently, cells were treated with 20 nM Taxol to inhibit microtubule polymerization and were imaged within 30 min of Taxol addition^52^. Photoconversion of a small subpopulation of microtubules was performed by 6 s exposure of 405 nm light from a Heliophor light source (89 North) constrained by a diaphragm. Photoconverted microtubules were imaged for at least 5 min with a 60-s interval using an inverted Nikon Eclipse U2000 microscope with a Yokogawa CSU10 spinning disk confocal head and Evolve EMCCD (Photometrics). Cells were photoconverted and imaged in groups of five using Nikon Elements software and a motorized stage. After photoconversion and imaging, BFP signal was imaged to confirm cells expressed WT or engineered kinesins. A custom Java-based Fiji plugin was developed to quantify microtubule sliding rates using the following methodology: time-lapse images of photoconverted microtubules were bleach-corrected and thresholded to detect microtubules. The initial photoconverted zone was identified in the first frame of each movie. The number of pixels corresponding to microtubules was measured in total or outside the initial zone for each frame of time-lapse movies. The motile fraction of microtubules was calculated for each frame by: %MF=microtubules^outside_initial_zone^/microtubules^total^. These values were then plotted against time and the slope of this graph was calculated for the initial linear section (identified by the highest *R*^2^ value of a linear regression, which contained at least four data points). This slope represents the gross microtubule sliding rate in each cell with the units, Change in % Motile Fraction * min^−1^.

### *In vitro* single-molecule motility assays

Flow chambers (∼10 μl volume) were constructed by attaching clean #1.5 cover glasses to microscope slides using double-sided adhesive tape. Microtubules were polymerized from a mixture of HiLyte647-labelled and unlabelled tubulins (Cytoskeleton) in BRB80 buffer (80 mM PIPES/KOH, pH 6.8, 1 mM MgCl_2_, 1 mM EGTA) in the presence of 1 mM GTP and 4 mM MgCl_2_ at 37 °C and stabilized by the addition of 20 μM Taxol. Microtubules were diluted in five volumes of P12 buffer (12 mM PIPES/KOH, pH 6.8, 2 mM MgCl_2_ and 1 mM EGTA) supplemented with 10 μM Taxol and immobilized in flow chambers by nonspecific adhesion. Flow chambers were subsequently incubated with 15 mg ml^−1^ bovine serum albumin in P12 buffer containing Taxol for 30–120 min at room temperature followed by Motility Mix containing 0.5–1.5 μl cell lysate in 30 μl of motility buffer (P12 buffer containing 17 μM Taxol, 1.7 mM MgCl_2_ and 1.7 mM dithiothreitol).

For each motor construct, motility assays in the absence and presence of inhibitor were carried out back-to-back. For ReAsH treatment, equal volumes of ReAsH-EDT_2_ or DMSO vehicle were added to the Motility Mix and incubated 30–60 min on ice. For B/B treatment, equal volumes of B/B homodimerizer or ethanol vehicle were added to the Motility Mix and incubated 30–60 min on ice. After incubation, 15 μl of P12 buffer containing 30 mg ml^−1^ bovine serum albumin, 2 mM ATP and an oxygen scavenger system (final concentrations 0.08 mg ml^−1^ catalase, 10 mM D-(+)-glucose, 0.2 mg ml^−1^ glucose oxidase) was added to yield a final volume of 50 μl. The solution was then introduced to the flow chamber, and the chamber was sealed with molten paraffin wax.

Imaging was carried out at room temperature on a Nikon Ti-E/B microscope equipped with a × 100, 1.49 NA oil-immersion TIRF objective, × 1.5 tube lens, three 20 mW diode lasers (488, 561, 640 nm) controlled via acousto-optical tunable filter (AOTF) (Agilent) and EMCCD detector (iXon X3 DU897, Andor). Images were acquired with 488 nm excitation, 100 ms exposure at 9.83 frames per second. A still image of the microtubule track was acquired via 640 nm excitation before and after live imaging.

Single-molecule tracking^53^ yielded motor trajectories that were filtered based on temporal (minimum life time≥0.5 s) and spatial (tracks fully overlap with the microtubule image) considerations. Motile events with a run length >250 nm were subjected to advanced trajectory analysis^54^. The run length was determined based on a start to end-point vector, rather than a sum of the instantaneous positions along the trajectory. Such run lengths are insensitive to positional noise and are hence suited to analysis of run lengths of slow events, for example, for the inhibited TC constructs. Run lengths and run speeds of trajectories were plotted as empirical cumulative distribution functions and fitted to exponential (run length) and normal (run velocity) distributions to extract the means of these values. Ninety-five percent confidence intervals were obtained by bootstrapping (*n* boots=2,000). Motile events were defined as motors landing and processively moving (>250 nm) along the microtubule, whereas immotile events were defined as a motor landing and staying attached to the microtubule for 2.5–10 s without movement (<250 nm). The number of events was normalized per micrometre of microtubule per minute.

### Code availability

Computer code used for data analysis is available upon request. Please contact K.J.V. (kjverhey@umich.edu) or M.F.E. (engelkem@umich.edu).

### Miscellaneous

Structural crystallography data were visualized with PyMOL (http://pymol.org).

## 

## Additional information

**How to cite this article:** Engelke, M. F. *et al.* Engineered kinesin motor proteins amenable to small-molecule inhibition. *Nat. Commun.* 7:11159 doi: 10.1038/ncomms11159 (2016).

## Supplementary Material

Supplementary Information Supplementary Figures 1-8, Supplementary Table 1

## Figures and Tables

**Table 1:** Microtubule gliding assay with TC-engineered *Dm*KHC constructs.

**Construct**	**Loop targeted**	**Putative function**	MT gliding results*	
			**Absence of FlAsH**	**Presence of FlAsH**
*Dm*KHC_TC(G30)	L1	ATP binding	597±12 nm s^−1^	No MT binding
*Dm*KHC_TC(G50)	L2	MT interaction	582±8 nm s^−1^	No MT binding
*Dm*KHC_TC(P61)	L3	ATP binding/hydrolysis	MT binding; no gliding	NA
*Dm*KHC_TC(G107)	L5	ATP binding/hydrolysis	MT binding; no gliding	NA
*Dm*KHC_TC(G174)	L8	MT interaction	MT binding; no gliding	NA
*Dm*KHC_TC(P312)	L14	MT interaction	113±7 nm s^−1^	106±9 nm s^−1^

MT, microtubule; NA, not applicable.

^*^Data are presented as the mean±s.e. of the mean. *n*≥30 microtubules for all conditions.
