# Supplementary material for: Engineered kinesin motor proteins amenable to small-molecule inhibition
Source: Nat Commun. 2016 Apr 5;7:11159. doi: 10.1038/ncomms11159 (PMC4822052; doi:10.1038/ncomms11159)
Supplement: Supplementary Information — Supplementary Figures 1-8, Supplementary Table 1 [file ncomms11159-s1.pdf]

a

Loop	Construct	Amino acid sequence
L1	*Dm*KHC-TC(G30)	NH$_2$-...E^{27}K^{28}A^{29}CCPG^{30}CCS^{31}K^{32}F^{33}...-COOH
L2	*Dm*KHC-TC(G50)	NH$_2$-...I^{48}A^{49}G^{50}CCPGCCK^{51}V^{52}Y^{53}...-COOH
L3	*Dm*KHC-TC(P61)	NH$_2$-...V^{58}F^{59}K^{60}CCP^{61}GCCN^{62}A^{63}S^{64}...-COOH
L5	*Dm*KHC-TC(G107)	NH$_2$-...G^{104}V^{105}I^{106}CCPG^{107}CCD^{108}S^{109}V^{110}...-COOH
L8	*Dm*KHC-TC(G174)	NH$_2$-...V^{172}K^{173}G^{174}CCPGCCA^{175}T^{176}E^{177}...-COOH
L14	*Dm*KHC-TC(P312)	NH$_2$-...C^{309}C^{310}S^{311}CCP^{312}GCCA^{313}S^{314}F^{315}...-COOH

b

```
                                                  L1                          L2
Dm KHC  1 msaereipaedsikvvcrfrplndseekagskfvvkfpnnveencisiagkvylfdkvfk 60
              ...|||.||||:|||||||::|...|.||:.||..  ||..:...||.|:||:|..
Rn KIF5C 1 ----madpaecsikvmcrfrplneaeilrgdkfipkfkg--eetvvigqgkpyvfdrvlp 54
```

c

Loop	Construct	Amino acid sequence
L1	*Rn*KIF5C-TC(G26)	NH$_2$-...R^{25}CCPG^{26}CCD^{27}K^{28}F^{29}I^{30}P^{31}...-COOH
L1	*Rn*KIF5C-TC(D27)	NH$_2$-...R^{25}G^{26}D^{27}CCPGCCK^{28}F^{29}I^{30}P^{31}...-COOH
L1	*Rn*KIF5C-TC(K28)	NH$_2$-...R^{25}G^{26}D^{27}K^{28}CCPGCCF^{29}I^{30}P^{31}...-COOH
L2	*Rn*KIF5C-TC(G42)	NH$_2$-...I^{41}G^{42}CCPGCCQ^{43}G^{44}K^{45}P^{46}Y^{47}V^{48}F^{49}D^{50}R^{51}...-COOH
L2	*Rn*KIF5C-TC(G44)	NH$_2$-...I^{41}G^{42}Q^{43}CCPG^{44}CCK^{45}P^{46}Y^{47}V^{48}F^{49}D^{50}R^{51}...-COOH
L2	*Rn*KIF5C-TC(K45)	NH$_2$-...I^{41}G^{42}Q^{43}G^{44}K^{45}CCPGCCP^{46}Y^{47}V^{48}F^{49}D^{50}R^{51}...-COOH

Supplementary Figure 1. Design of engineered kinesin-1 constructs. (**a**) TC-tagged *Dm*KHC motors. Listed are the surface loop (L) location of TC insertion site, the construct name, and the sequence at TC (CCPGCC) insertion site (one letter amino acid code). Superscript numbers denote amino acid position in the protein; NH$_2$, N-terminus; COOH; C-terminus. (**b**) Alignment of the N-terminal sequences of *Dm*KHC and *Rn*KIF5C using the Needleman- Wunsch algorithm (EMBL-EBI). A vertical line denotes identical amino acids, two dots highly similar amino acids, and one dot similar amino acids in the sequence comparison. (**c**) TC-tagged *Rn*KIF5C motors. Listed are the surface loop (L) location of TC insertion site, the construct name, and the sequence at TC (CCPGCC) insertion site (one letter amino acid code).

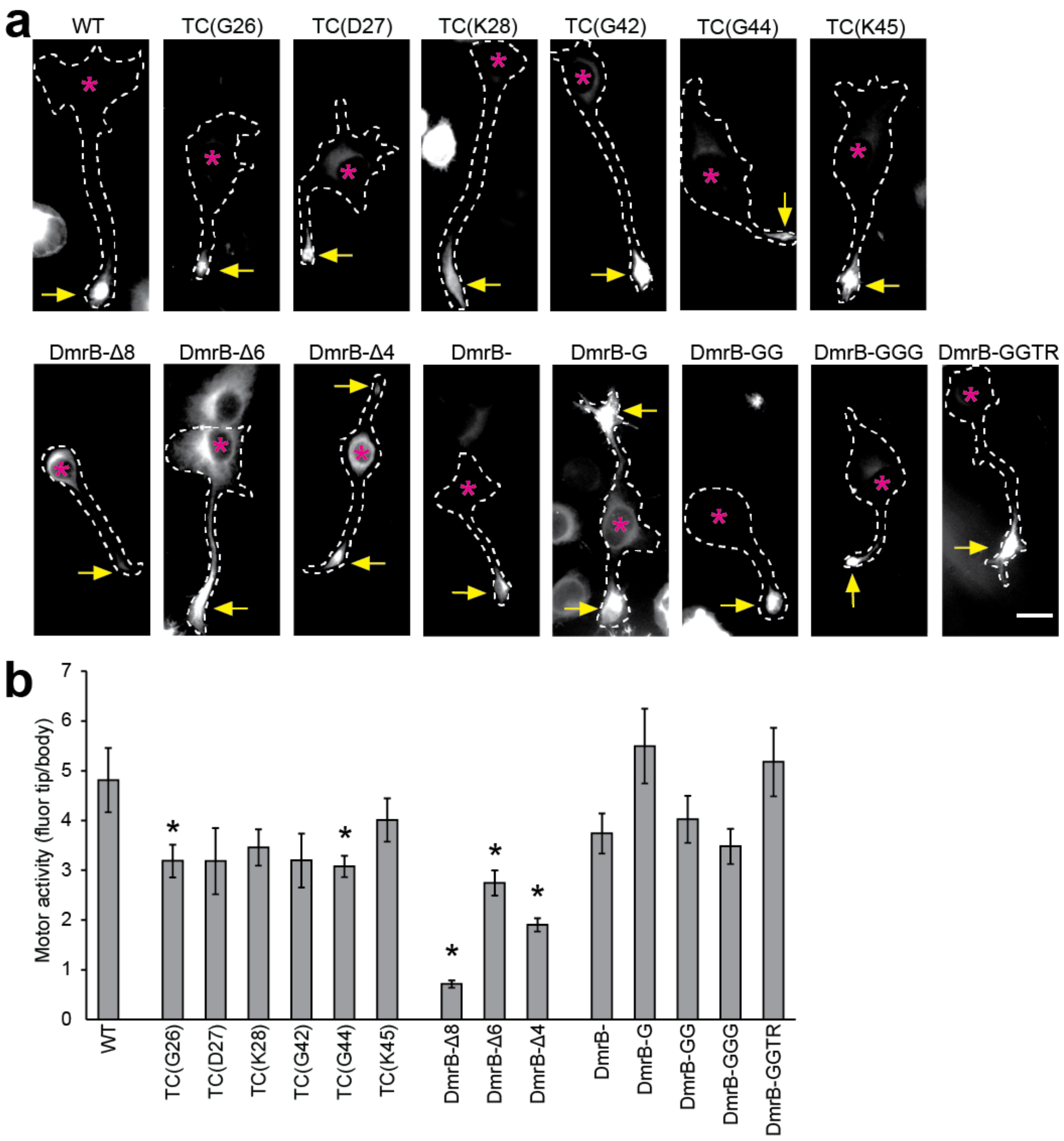

Supplementary Figure 2. Neurite tip accumulation assay. CAD cells transfected with mCit-tagged *Rn*KIF5C(1-559) motor constructs were serum-starved to induce the formation of axon-like neurites. Two days later, cells were fixed and imaged. Scale bar; 15 µm. (**a**) Representative images showing the subcellular distribution of the indicated motor constructs. (**b**) Quantification of tip accumulation. The data are reported as the ratio of mean fluorescence in the neurite tip over the mean fluorescence near the cell body (n ≥ 19 cells each). A high ratio indicates an active *Rn*KIF5C(1-559) motor whereas a low ratio indicates an inactive motor. Error bars, SEM; * $p<0.05$ as compared to WT, calculated with a heteroscedastic, two-tailed t test.

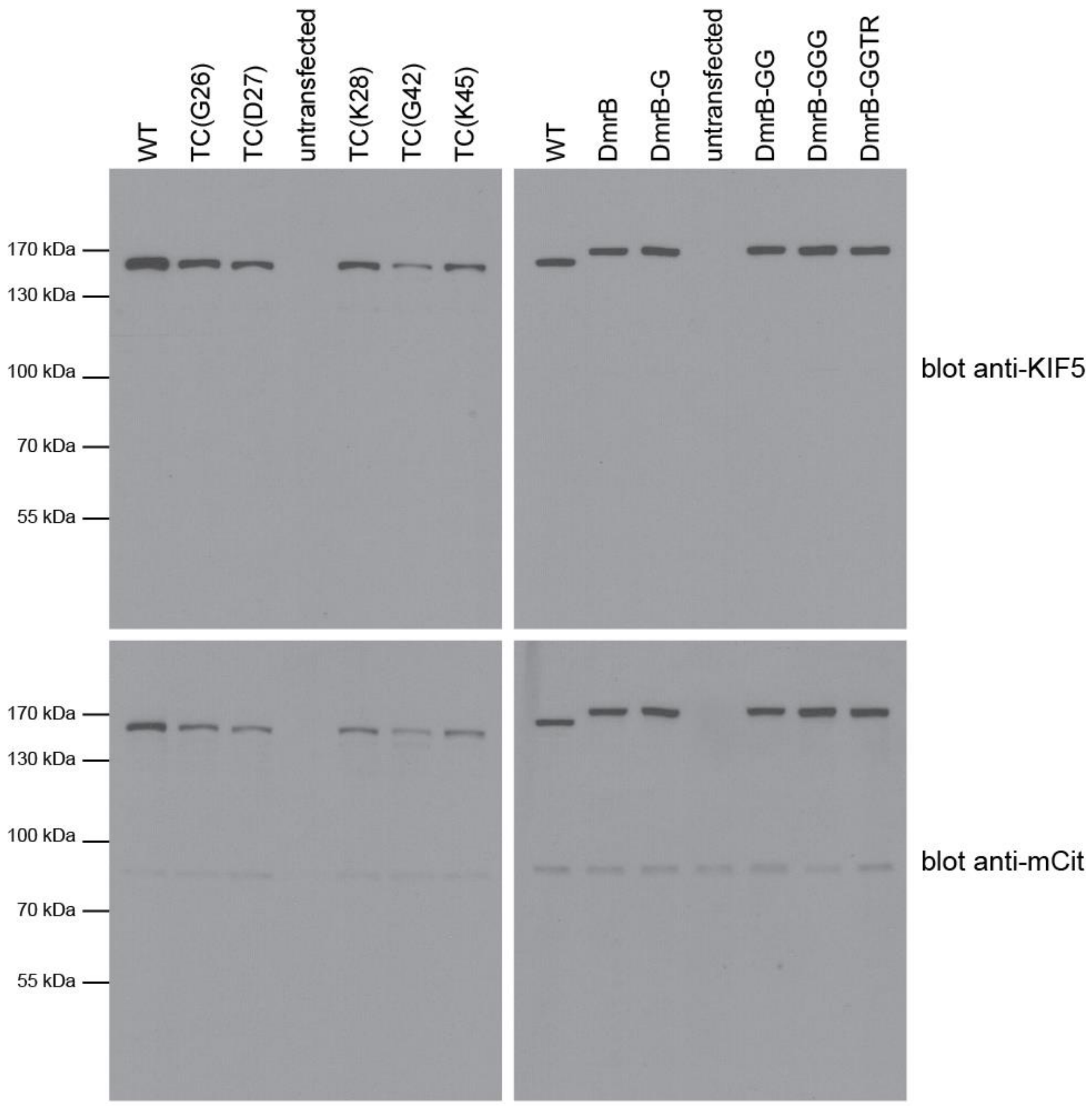

Supplementary Figure 3. Expression of engineered *Rn*KIF5C motor constructs in COS7 cells. Lysates of cells expressing the indicated TC- and DmrB-tagged constructs were immunoblotted with antibodies to kinesin-1 (H2; Covance MAB1614) and mCitrine (A6455; Invitrogen) as indicated. WT, wild type.

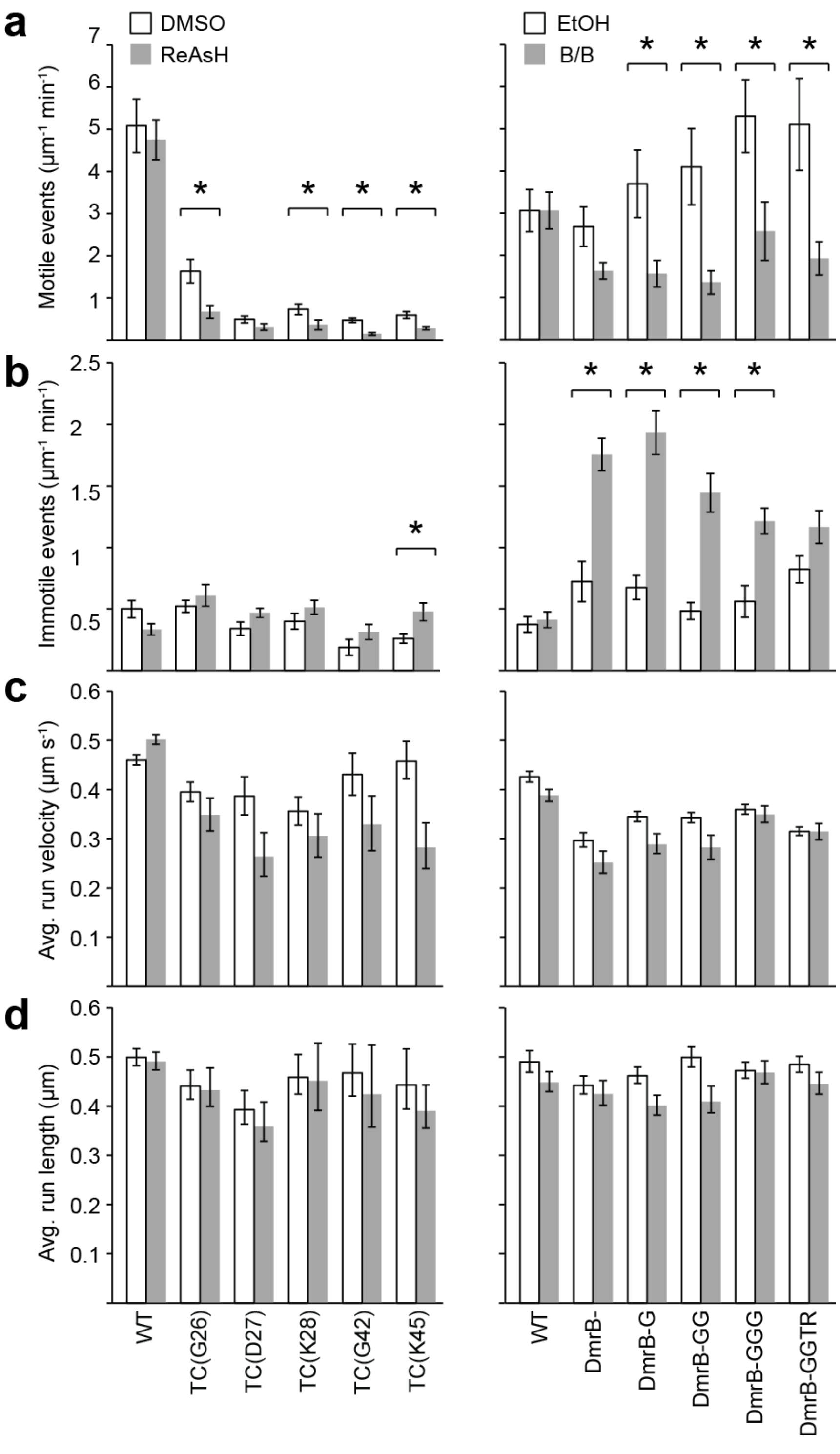

Supplementary Figure 4. Single molecule motility properties of the engineered motors in the absence (EtOH, DMSO) or presence (20 µM ReAsH or 1.5 µM B/B) of the respective inhibitor. (**a,b**) The frequency of (**a**) motile and (**b**) immotile events for each construct and treatment. Error bars, SEM; * $p<0.05$; heteroscedastic, two-tailed t test comparing motility in the absence and presence of drug. For the motile events, the mean (**c**) velocity and (**d**) run length is shown. Error bars indicate 95% confidence interval obtained by bootstrapping.

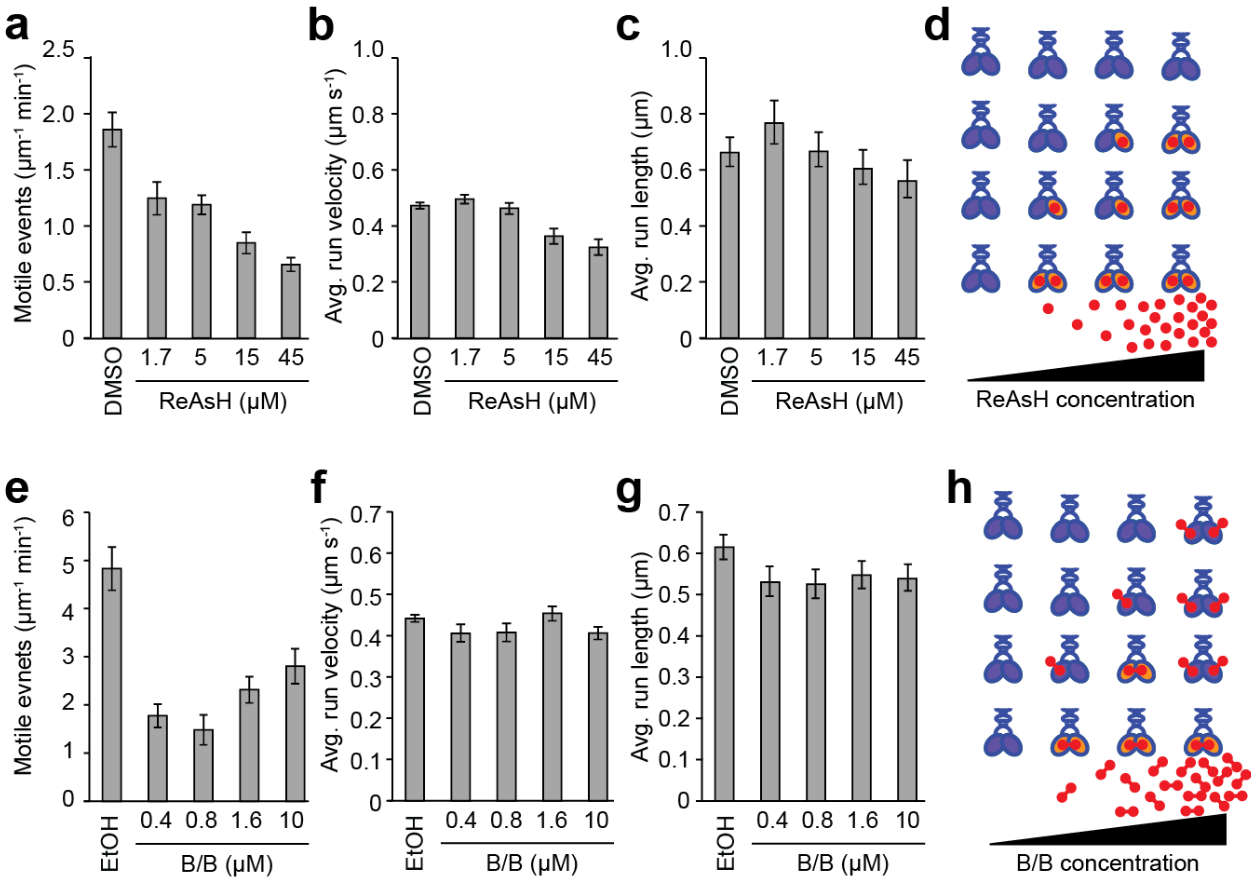

Supplementary Figure 5. Dose-response of motor inhibition in single molecule motility assays. (**a-c**) Lysates of COS7 cells expressing TC(G26) *Rn*KIF5C(1-559)-3xmCit motors were incubated in parallel for 30 min with DMSO control or the indicated concentrations of ReAsH and then imaged by TIRF microscopy. (**e-g**) Lysates of COS7 cells expressing DmrB-GG *Rn*KIF5C(1-559)-3xmCit motors were incubated in parallel for 30 min with EtOH control or the indicated concentrations of B/B and then imaged by TIRF microscopy. The average (**a, e**) number of motile events, (**b, f**) run velocity, and (**c, g**) run length for each condition was determined from 9 time-lapse movies over three independent experiments ($n \geq 136$ events per condition). Error bars represent **(a, e**) SEM or (**b-c; f-g**) the 95% confidence interval from bootstrapping. (**d,h**) Models for the concentration-dependent effects of (**d**) ReAsH or (**h**) B/B on the motility of the respective engineered motor. (**d**) Increasing ReAsH (red circle) concentrations result in a corresponding increase in the percentage of inhibited (orange) motor heads. (**h**) At low B/B (red dumbbell) concentrations, the B/B inhibitor cross-links the two DmrB-tagged motor heads of a dimeric motor, consequently inhibiting processive motility. However, at high B/B concentrations, separate B/B inhibitor molecules can bind to each of the DmrB-tagged motor heads of a dimeric motor, which does not result in crosslinking and thus does not inhibit processive motility.

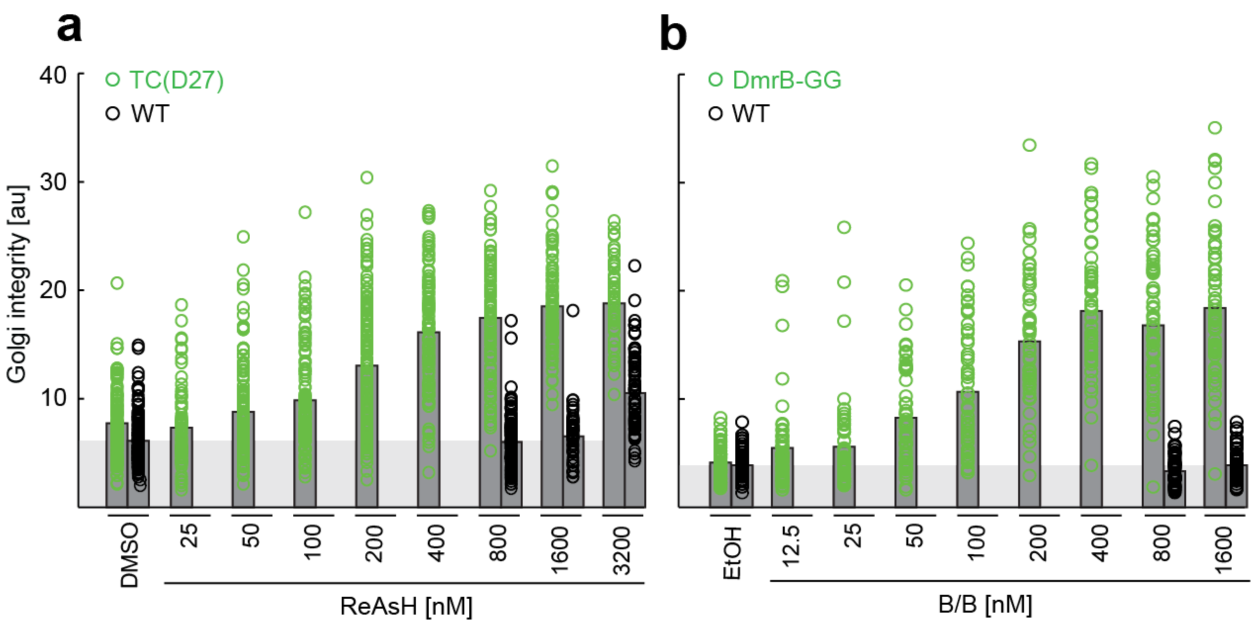

Supplementary Figure 6. Dose-response of motor inhibition in the Golgi dispersion assay. COS7 cells were transfected with plasmids for expressing Golgi-targeted (**a**) WT or TC(D27) kinesin-1 motors or (**b**) WT or DmrB-GG kinesin-1 motors and were treated with (**a**) DMSO vehicle control or the indicated concentrations of ReAsH or (**b**) EtOH vehicle control or the indicated concentrations of B/B. 14 h later, the cells were fixed and stained with DAPI and an antibody to Giantin and the Golgi integrity was measured on a cell-by-cell basis. Low values represent a dispersed Golgi complex (motor active) whereas high values represent a compact, perinuclear Golgi complex (motor inhibited). Each circle represents the Golgi integrity of an individual cell. Gray bars represent means of at least 49 cells across one or two independent experiments.

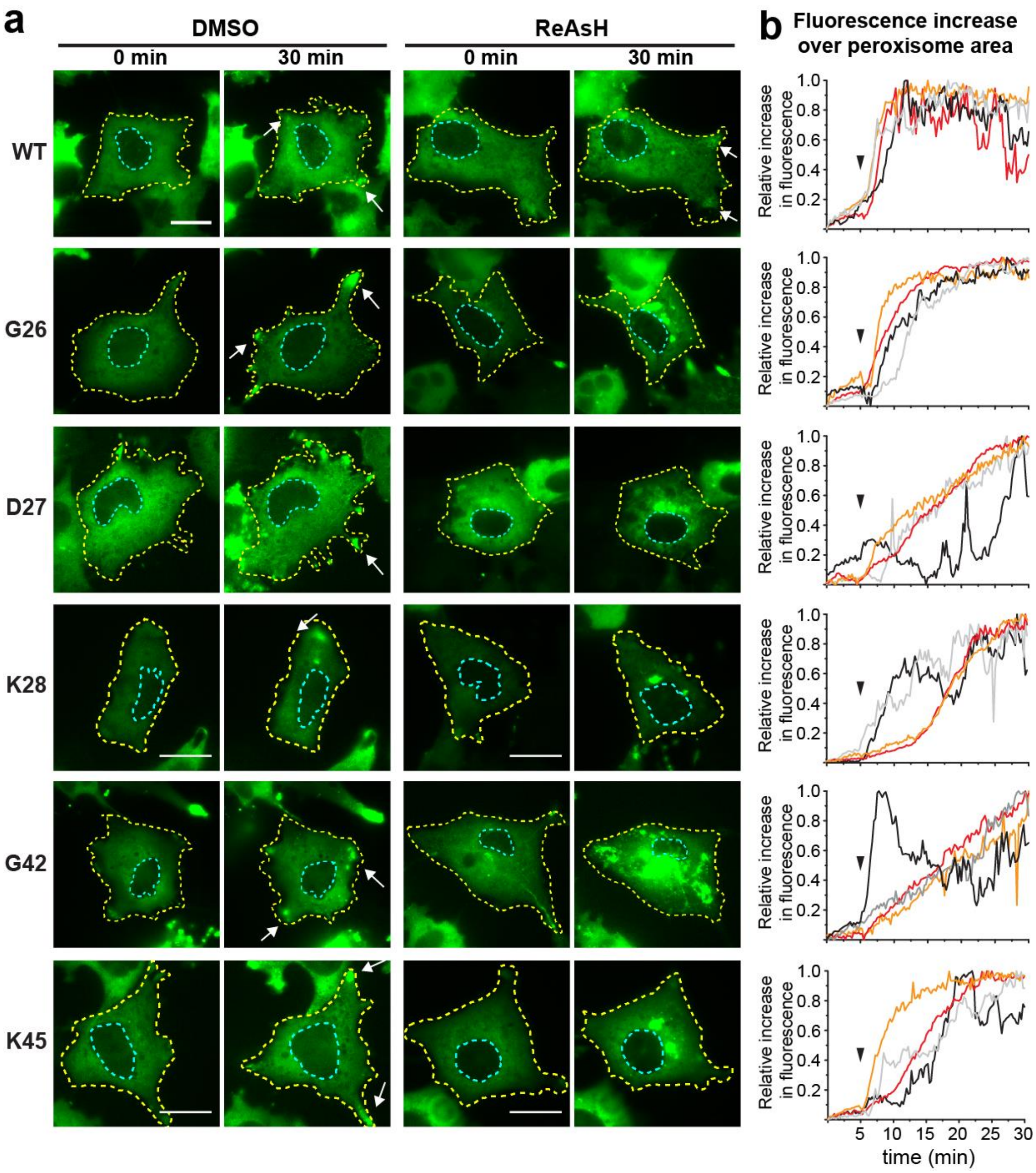

Supplementary Figure 7. Motor recruitment to the peroxisome surface during the peroxisome redistribution assay. Cells expressing the indicated WT or TC-tagged motor constructs were treated with DMSO (vehicle control) or 200 nM ReAsH for 30 min. Cells were then imaged for 30 min with rapamycin added 5 min after the start of imaging to induce motor recruitment to the peroxisome surface. (**a**) Representative images of the *Rn*KHC(1-559)-mCit-DmrC fluorescence at 0 and 30 min of rapamycin addition. Yellow dotted line, cell periphery; blue dotted line, nucleus. Arrows indicate the accumulation of *Rn*KIF5C-labeled peroxisomes in the periphery of the cell. Scale bar, 20 µm. (**b**) Time course of increase in mCit fluorescence over the peroxisome area in the presence of DMSO (black and light grey lines) or ReAsH (red and orange lines). Arrowhead, time of rapamycin addition. The data are presented as the relative increase in mCit fluorescence over the peroxisome area normalized to initial and maximal fluorescence of 0 and 1.0, respectively.

a

Loop	Construct	Amino acid sequence
L1	*Rn*KIF1A-TC(D23)	NH_2-...S^{21}R^{22}D^{23}CCPGCCS^{24}K^{25}C^{26}I^{27}...-COOH
L1	*Rn*KIF1A-TC(K25)	NH_2-...R^{22}D^{23}S^{24}K^{25}CCPGCCC^{26}I^{29}I^{30}...-COOH
L2	*Rn*KIF1A-TC(K41)	NH_2-...V^{38}N^{39}P^{40}K^{41}CCPGCCQ^{42}P^{43}K^{44}E^{45}...-COOH
L2	*Rn*KIF1A-TC(S51)	NH_2-...K^{48}S^{49}F^{50}S^{51}CCPGCCF^{52}D^{53}Y^{54}S^{55}...-COOH

b

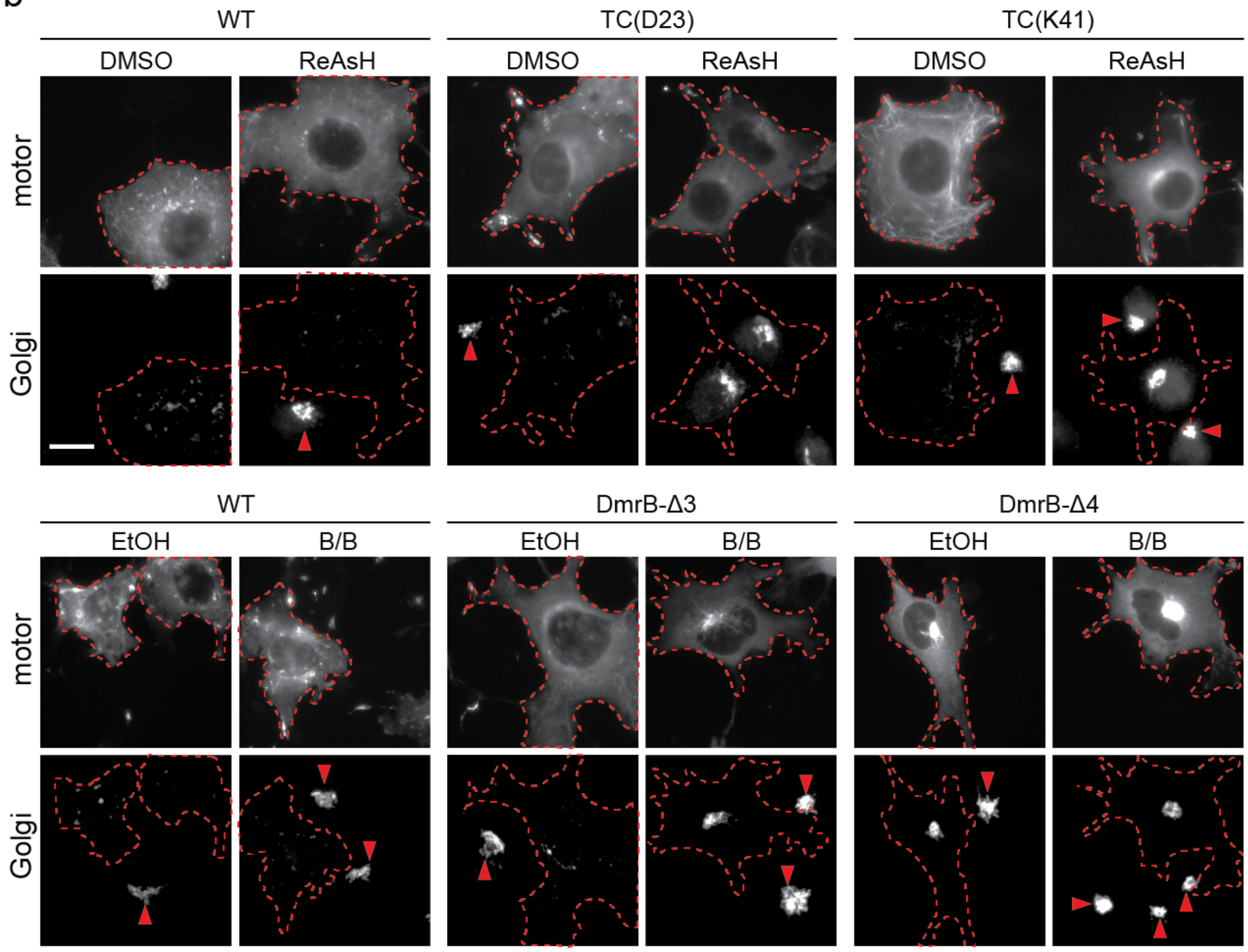

Supplementary Figure 8. Engineering of inhibitable kinesin-3 constructs. (**a**) Schematic of TC-tagged *Rn*KIF1A constructs. Listed are the surface loop (L) location of the TC insertion site, the construct name, and the sequence at the TC (CCPGCC) insertion site (one letter amino acid code). Superscript numbers denote amino acid position in the protein; NH_2, N-terminus; COOH; C-terminus. (**b**) Representative images of the Golgi dispersion assay for engineered *Rn*KIF1A constructs. COS7 cells were transfected with the indicated constructs in the presence (400 nM ReAsH; 1.5 µM B/B) or absence (DMSO; EtOH) of inhibiting drug. 12-14 h post transfection, cells were fixed and nuclei were stained with DAPI and the Golgi was stained with an antibody against Giantin. Red dotted lines indicate outline of transfected cells; red arrowheads indicate compact Golgi apparatus in non-transfected cells. Scale bar, 15 µm.

Supplementary Table 1: Primer sequences for generating TC-tagged *Dm*KHC(1-560)-EGFP-His6 constructs.

Construct	PCR Method	Primer Sequences
TC(G30)	SOE*	FOR #1: TTTCCCTCTAGAAATAATTTTG
		REV #1: TTGGAGCAACAGCCTGGGCAACAGGCCTTCTCTTCGCTGTC
		FOR #2: AGGCCTGTTGCCCAGGCTGTTGCTCCAAGTTCGTGGTCAAGTTC
		REV #2: CTCAAGCTTCTCCACCTTAC
TC(G50)	SOE*	FOR #1: TTTCCCTCTAGAAATAATTTTG
		REV #1: ACCTTGCAACATCCTGGGCAACAGCCCGCTATGGATATGCAG
		FOR #2: GGGCTGTTGCCCAGGATGTTGCAAGGTGTATTTGTTCGACAAGG
		REV #2: CTCAAGCTTCTCCACCTTAC
TC(P61)	QuikChange	FOR step 1: GACAAGGTCTTCAAATGTTGCCCGAATGCATCCC
		REV step 1: GGGATGCATTCGGGCAACATTTGAAGACCTTGTC
		FOR step 2: CAAATGTTGCCCGGGATGCTGTAATGCATCCCAGG
		REV step 2: CCTGGGATGCATTACAGCATCCCGGGCAACATTTG
TC(G107)	QuikChange	FOR step 1: GAGGGCGTGATCTGTTGCCCTGGGGACTCCGTAAAAC
		REV step 1: GTTTTACGGAGTCCCCAGGGCAACAGATCACGCCCTC
		FOR step 2: CTGTTGCCCTGGGTGTTGCGACTCCGTAAAACAG
		REV step 2: GTCGCAACACCCAGGGCAACAGCTGTTTTACGGA
TC(G174)	QuikChange	FOR step 1: GTACGTCAAGGGCTGTTGCCCGGCTACGGAACGG
		REV step 1: CCGTTCCGTAGCCGGGCAACAGCCCTTGACGTAC
		FOR step 2: GCTGTTGCCCGGGATGCTGTGCTACGGAACG
		REV step 2: CGTTCCGTAGCACAGCATCCCGGGCAACAGC
TC(P312)	QuikChange	FOR step 1: CATCTGCTGCTCTTGTTGCCCAGCCAGTTTCAAC
		REV step 1: GTTGAAACTGGCTGGGCAACAAGAGCAGCAGATG
		FOR step 2: GCTCTTGTTGCCCAGGATGCTGTGCCAGTTTCAACG
		REV step 2: CGTTGAAACTGGCACAGCATCCTGGGCAACAAGAGC

*SOE, splice by overlap extension
